# A Micromechanical Transmitter with Only One BAW Magneto-Electric Antenna

**DOI:** 10.3390/mi13020272

**Published:** 2022-02-08

**Authors:** Si Chen, Junru Li, Yang Gao, Jianbo Li, Hongmei Dong, Zhijun Gu, Wanchun Ren

**Affiliations:** 1 School of Information Engineering, Southwest University of Science and Technology, Mianyang 621010, China; chensi@mails.swust.edu.cn (S.C.); gaoy@swust.edu.cn (Y.G.); lijb@mails.swust.edu.cn (J.L.); donghongmei@mails.swust.edu.cn (H.D.); guzj@mails.swust.edu.cn (Z.G.); rwch_qw@163.com (W.R.); 2 College of Optoelectronic Engineering, Chongqing University, Chongqing 400044, China; 3 Robot Technology Used for Special Environment Key Laboratory of Sichuan Province, Mianyang 621010, China

**Keywords:** bulk acoustic wave (BAW), magnetoelectric transducer, antenna, transmitter, implantable medical devices (IMDs), resonance modes, frequency modulation, radiation power, FEA

## Abstract

Implantable medical devices have been facing the severe challenge of wireless communication for a long time. Acoustically actuated magnetoelectric (ME) transducer antennas have attracted lots of attention due to their miniaturization, high radiation efficiency and easy integration. Here, we fully demonstrate the possibility of using only one bulk acoustic wave (BAW) actuated ME transducer antenna (BAW ME antenna) for communication by describing the correspondence between the BAW ME antenna and components of the traditional transmitter in detail. Specifically, we first demonstrate that the signal could be modulated by applying a direct current (DC) magnetic bias and exciting different resonance modes of the BAW ME antenna with frequencies ranging from medium frequency (MF) (1.5 MHz) to medium frequency (UHF) (2 GHz). Then, two methods of adjusting the radiation power of the BAW ME antenna are proposed to realize signal amplification, including increasing the input voltage and using higher order resonance. Finally, a method based on electromagnetic (EM) perturbation is presented to simulate the transmission process of the BAW ME antenna via the finite element analysis (FEA) model. The simulation results match the radiation pattern of magnetic dipoles perfectly, which verifies both the model and our purpose.

## 1. Introduction

Wireless implantable medical devices (IMDs) have sparked a new wave of research on biomedical technology [1,2,3]. However, the problem of wireless communication has, for a long time, been an arduous challenge in the development of IMDs [4,5,6]. One of the main reasons is that the sizes of the antennas used for signal transmission are too large to be integrated in IMDs [7,8,9]. In addition, the existing IMDs require an additional power source, which could further increase the size of the entire IMDs [10,11,12]. A number of transducers used to provide power sources in IMDs have successfully reduced the size to the scale of the diameter of a piece of human hair [13,14,15,16]. However, the transducing efficiency of the current prototypes is too low to apply. Therefore, new antenna technologies need to be developed to solve the problem of wireless communication of IMDs in the Industrial, Scientific and Medical (ISM) band. A novel bulk acoustic wave (BAW) actuated magnetoelectric (ME) antenna was proposed theoretically in 2015, the efficiency bandwidth product of which was close to Chu’s limit [17]. In 2017, Northeastern University successfully prepared this antenna and found that it can work comfortably in the ISM band [18]. The size of the antenna can be greatly reduced because the electromagnetic (EM) resonance of the traditional antenna is replaced by the acoustic resonance of the BAW ME antenna. In addition, the BAW ME antenna is also an EM energy harvester from the view of receiving signals [19]. The magnetostrictive layer in the BAW ME antenna is used for sensing magnetic signals, and the piezoelectric layer is used for generating charges. This means that no additional power source is required to provide the energy supply of IMDs, which further reduces the size of the IMDs.

In fact, Jensen et al. successfully used a carbon nanotube antenna to play music back in 2007, which gives us a great inspiration [20]. The BAW ME antenna may have a more profound meaning, that is, using a single BAW ME antenna can also achieve wireless communication. If this view can be confirmed, it will have important implications for the definition of potential application scenarios of the BAW ME antenna. To complete the verification, we considered a solution using finite element analysis (FEA) simulation under the existing conditions, since Xu et al. recently constructed an EM radiation model of ME composites using FEA software [21]. However, the existing commercial FEA software hardly involves magnetization dynamics and magnetic spin simulations, so it is difficult to build a radiation model of the BAW ME antenna to verify its signal transmission behavior.

In this article, we explain and demonstrate how to achieve the wireless transmission of signals using only one BAW ME antenna. We first review the basic components and working principles of the traditional transmitter. For the benchmarking modulator, three kinds of modes of vibration are used to realize frequency modulation. Regarding the benchmarking power amplifier, the influence of the input voltage and higher order resonant frequency on the radiated power is demonstrated, which enables the signal to be amplified. Based on EM perturbations and multiphysics coupling, we used FEA simulation to analyze the whole operating process for the BAW ME transmitter. The focus of this simulation was to evaluate the near-field radiation of the BAW ME antenna by using the amplitude and distribution of the EM field with regular variation in the EM environment. We also compared the simulated data with the near-field pattern of an ideal magnetic dipole to verify the simulation scheme based on EM perturbation.

## 2. BAW ME Transmitter

### 2.1. Fundamentals of Radio Transmitters

**Figure 1:**
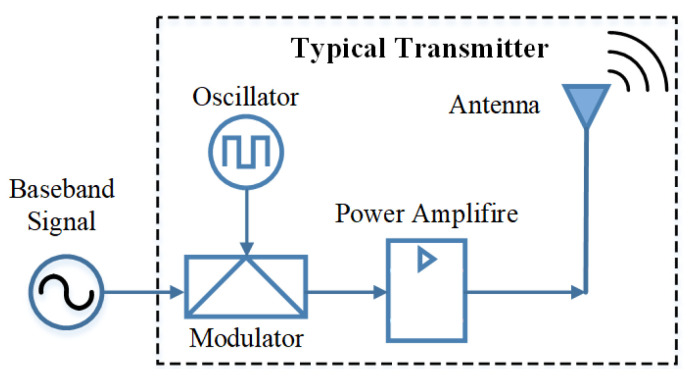
Block diagram for a typical transmitter. The four essential components can be replaced by a BAW ME antenna.

A transmitter is an electrical system that can transmit a signal according to a certain frequency. A typical transmitter includes four essential components: an oscillator, a modulator, an amplifier and an antenna, as shown in Figure 1. The modulated signal is amplified by a power amplifier and radiated through the antenna into free space.

**Figure 2:**
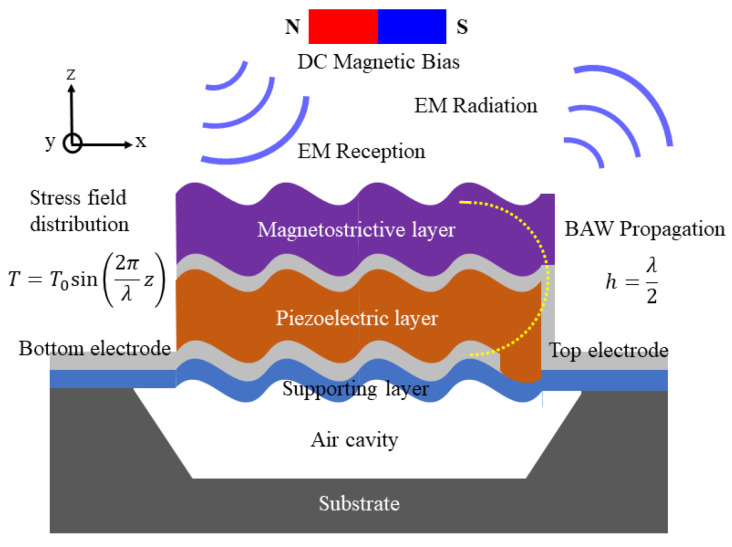
Schematic of the BAW ME antenna in resonance. The direct current (DC) magnetic bias is used to apply prestress, which can be achieved using a DC wire.

The four essential components of a transmitter can be replaced by just one BAW ME antenna, as shown in Figure 2. The piezoelectric layer and electrodes form a BAW resonator to power the antenna. The magnetostrictive layer, the radiation source of the antenna, is coupled with the piezoelectric layer through stress/strain. From the perspective of the operating principle, the actuating source of the antenna is the BAW resonator, and the radiation source is magnetostrictive film integrated on the resonator. When the alternating voltage that is consistent with the resonant frequency is applied to the top and bottom electrodes of the BAW resonator, the BAW resonator generates acoustic resonance to induce magnetization oscillation of the magnetostrictive film [22]. Finally, dynamic flux forms and an EM wave transmits.

### 2.2. Oscillator and Modulator

The BAW ME antenna is essentially a mechanical oscillator (also known as an acoustic resonator); this state is achieved by alternating the voltages applied to both sides of the piezoelectric layer.

**Figure 3:**
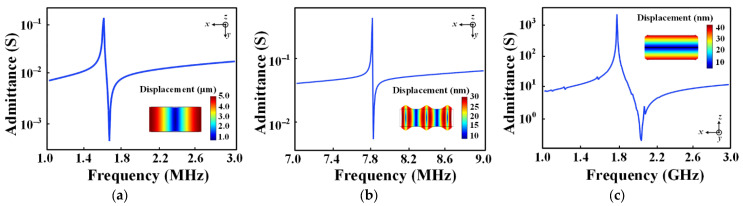
Admittance characteristic curve of the BAW resonator corresponding to the three resonant modes. The inset is the displacement field distribution at different resonance modes: (**a**) longitudinal resonance mode; (**b**) contour resonance mode; (**c**) thickness resonance mode.

The BAW resonator can be designed to operate in different vibration modes, including longitudinal, contour and thickness resonance mode. According to Equation (1), the frequency calculation for the shear and longitudinal wave, different resonant modes correspond to different resonant frequencies *f*.

(1) $$f=\{\begin{array}{c} \frac{1}{2L}\sqrt{\frac{Y}{\rho }}\\ \frac{1}{2h}\sqrt{\left(c_{33}+\frac{d_{33}}{e_{33}}\right)/\rho } \end{array},$$

where *L* is the length or width of the materials, *h* is the thickness of the materials, *Y* is the Young’s modulus of the materials, *ρ* is the mass density of the materials, and *c*_33_, *d*_33_ and *e*_33_ are the elastic, piezoelectric and dielectric coefficients of the materials, respectively. Therefore, frequency tunableness in the BAW ME antenna could be accomplished by exciting different resonance modes of the BAW resonator. Its operating frequency ranges from medium frequency (MF) to ultra-high frequency (UHF), entirely depending on its size. As shown in Figure 3, the three vibration modes of the BAW resonator can be identified according to the admittance characteristics of the FEA simulation at different frequencies. It can be seen that the BAW resonator frequency spans a wide range including about 1.5 MHz, 7.8 MHz and 2 GHz, which is conducive to the frequency selection of the BAW antenna at large scales. In order to achieve frequency modulation, a DC magnetic bias is applied to change the tension of the active region. The signal can be applied to this bias to modulate the resonant frequency. The film is prestressed, and the resonant frequency (also known as the carrier) changes with the information signal.

### 2.3. Power Amplifier (PA)

The function of the PA is to enhance the antenna radiation power by enhancing the signal amplitude. For the BAW ME antenna, there are two ways to control the radiated power effectively. Taking the BAW ME antenna working in thickness resonance mode as an example, our team [23] proposed a method for calculating the average radiated power of the BAW ME antenna considering the eddy current loss, which describes the relationship between the average radiated power and the design parameters, as shown in (2). According to this relation, schemes can be made to improve the average radiated power.

(2) $$P_{rad}=\frac{\omega^{2}h^{2}d_{33}^{2}}{2\eta }\iint_{s}{\left|T\right|}^{2}ds\approx \frac{\omega^{2}h^{2}d_{33}^{2}{\left|T\right|}^{2}A}{2\eta },$$

where *η* is the wave impedance of the free space, *d*_33_ is the piezomagnetic coefficient of the magnetostrictive layer in the z-direction, *ω* is the angular frequency, which represents the resonant frequency of the device, *h* is the thickness of the magnetostrictive layer, *A* is the area of the active region, and ***T*** is the longitudinal stress tensor. After designing and fabricating the BAW ME antenna, the structure and material parameters in Equation (2) are fixed and cannot be changed. Only the two parameters of stress ***T*** and frequency *ω* can be used to control the average radiated power. Next, we will demonstrate two ways to increase the internal stress and operating frequency of the device to achieve power amplification without additional PA.

**Figure 4:**
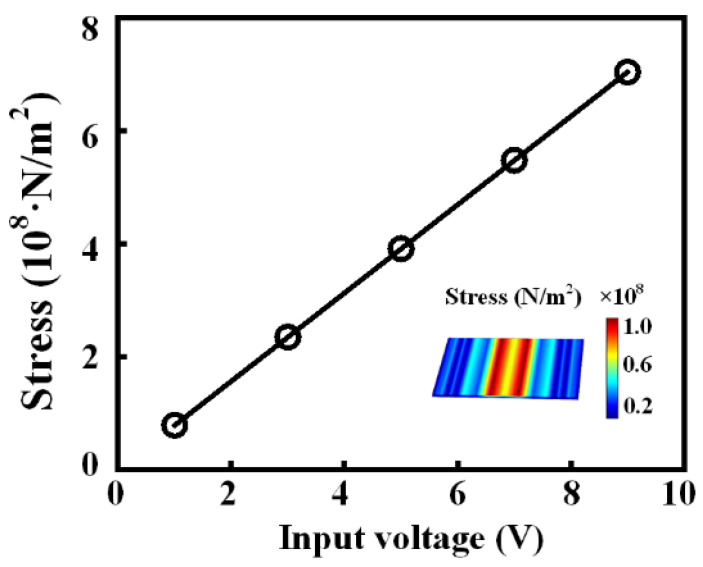
Relationship between stress and input voltage at resonance. The inset shows the stress field distribution of the magnetostrictive layer at particular input voltages. The size of the antenna is 100 × 50 × 1 µm^3^, and the resonant frequency is about 2.6 GHz.

The first method we propose is to increase the stress by increasing the excitation voltage. We demonstrate this process by FEA simulation, as shown in Figure 4. The result shows that the internal stress of the BAW ME antenna increases linearly with the amplitude of the input voltage, which means that increasing the excitation voltage within the power handing of the BAW resonator can easily achieve the purpose of increasing the radiated power.

**Figure 5:**
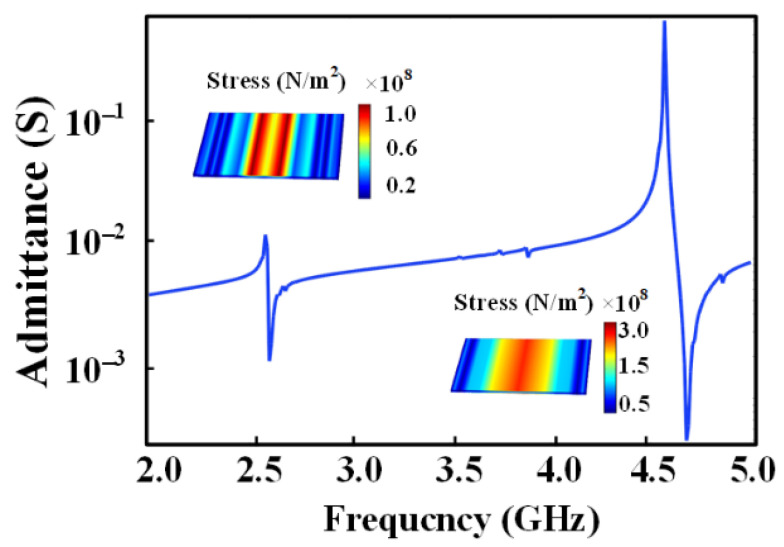
Admittance characteristic curve of the BAW ME antenna. An excitation voltage of 1 V is applied to obtain the admittance curve in the first-order and second-order resonant frequency. The inset is the stress field distribution of the antenna at different resonant frequencies.

The second method we propose is to increase the stress by using the higher order resonance characteristics of the BAW resonator, which is a solution that raises both the stress and the frequency. However, it is worth noting that the effect of ferromagnetic resonance (FMR) on the performance of the antenna should be considered [24], which means that the resonance frequency should be controlled below the FMR frequency. In order to investigate whether the higher order resonant frequency would have an effect on the stress, we used FEA simulation to analyze the harmonic response of the antenna and plotted the stress field distribution at the resonant frequency. As shown in Figure 5, the average stress of the antenna at the first-order resonant frequency is about 70 MPa, while that at the second-order resonant frequency is about 200 MPa. The results confirm that the radiation power of the antenna can be improved by using higher order resonance frequencies. In addition, it was previously reported that by keeping the FMR frequency of the magnetostrictive layer consistent with the resonance frequency of the BAW ME antenna, the radiation power of the device can be further improved [25].

In short, all the four essential components of a typical transmitter can be compactly and efficiently replaced by the BAW ME antenna to realize wireless communication.

## 3. Simulation of Transmission Process

This section focuses on the simulation of the transmitting process of the BAW ME antenna, which is not a small challenge, since the transmitting process of the BAW ME antenna involves magnetic spin. However, most existing commercial modeling software applications are limited to simulating simple magnetic behaviors such as magnetostriction. The response to magnetic spin that is usually achieved by magnetization dynamics is not available in FEA software. Therefore, there is no reference report on modeling the transmitting process of the BAW antenna using FEA. However, it is well known that the aperture EM field of the material’s surface is easy to obtain via FEA simulation based on the constitutive relation of ME material as shown in (3).

(3) $$\left[\begin{array}{c} S\\ E,H \end{array}\right]=\left[\begin{array}{cc} s_{D,B} & \frac{d_{E,H}}{\varepsilon ,\mu }\\ \frac{d_{E,H}}{\varepsilon ,\mu } & \frac{1}{\varepsilon ,\mu } \end{array}\right]\left[\begin{array}{c} T\\ D,B \end{array}\right],$$

where ***S*** and ***T*** are the stress and strain tensors satisfying the boundary conditions, respectively, ***E*** and ***H*** are the vectors of the electric and magnetic field intensity, respectively, ***D*** and ***B*** are the vectors of the electric and magnetic flux density, respectively, *ε* and *μ* are the dielectric constant and permeability of the film, respectively, under a no-stress condition, *s_D,B_* are the compliance constants of the piezoelectric and magnetostrictive layers, respectively, and *d_E,H_* are the strain constants of the piezoelectric and magnetostrictive layers, respectively. This aperture EM field will inevitably act as a perturbation source to break the uniform and balanced EM environment around it. If the amplitude and distribution of EM field change regularly in this EM environment, this variation is used to evaluate the near-field radiation of the BAW antenna.

Therefore, a simulation scheme of near field EM perturbations is proposed, which also solves the problem of huge physical scale differences between models and reality. The size of the BAW ME antenna is on the micron scale and the far field distance (*r* >> *λ*, *λ* is the EM wavelength) is over 0.1 m even at the resonant frequency of gigahertz. Therefore, extrapolating the far-field solution from the near-field distribution derived from EM perturbations is the only feasible FEA simulation method.

Specifically, the whole FEA model of the BAW ME antenna, including the magnetostrictive layer, piezoelectric layer, and air domain, is constructed to simulate the acoustic resonance, inverse ME effect and EM perturbations. The BAW ME antenna of 100 × 50 × 1 µm^3^ in size is located in the central point of the air domain, whose first-order resonance frequency is about 2.6 GHz. The radius of the air domain is set to 1000 µm. Then, the 3D model is solved in the frequency domain by coupling the electrostatic, solid mechanical and magnetic fields, as shown in (4)–(6), respectively.

(4) $$\mathrm{Electrostatic}\;\mathrm{field}:\;\{\begin{array}{c} \nabla \cdot D=\rho_{V}\\ E=-\nabla V\\ D=\varepsilon E+P \end{array},$$

(5) $$\mathrm{Solid}\;\mathrm{mechanical}\;\mathrm{field}:\;\{\begin{array}{c} \nabla \cdot T=\rho \frac{\partial^{2}u}{\partial t^{2}}\\ S=\frac{1}{2}\left(\nabla u+\nabla u^{T}\right) \end{array},$$

(6) $$\mathrm{Magnetic}\;\mathrm{field}:\;\{\begin{array}{c} \nabla \times H=J\\ B=\nabla \times A\\ E=-j\omega A \end{array},$$

where ∇ is the Laplace operator, $\rho_{V}$ is the charge density, *ε* is the permittivity, ***P*** is the polarization vector, ***u*** is the displacement vector, ***J*** is the ampere density vector, and ***A*** is the magnetic vector potential. The air domain is used for simulating the near-field of the antenna and analyzing the magnetic flux density of different positions near the antenna. Under the action of EM perturbations, the change rule of the flux density near the antenna can be detected. Of course, this is not used to simulate the EM radiation of the antenna by FEA, but to replace the radiation with EM perturbation at different positions, which means that the simulated EM field represents the near-field of the BAW ME antenna that does not propagate.

**Figure 6:**
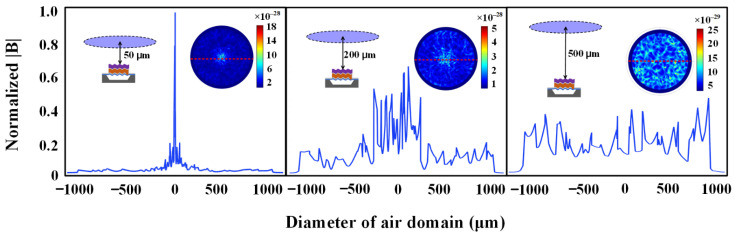
Simulation of near field normalized flux density. The insets show the distribution of the magnetic flux density on the horizontal plane 50 μm, 200 μm and 500 μm away from the antenna.

When the BAW ME antenna operates at the first-order resonant frequency, the amplitudes and distributions of magnetic flux density ***B*** in the range of 0~900 μm from the antenna along the z-direction are obtained. As shown in Figure 6, the magnetic flux density distributions along the red dotted line tracking on the horizontal plane in the insets at distances of 50 μm, 200 μm and 500 μm away from the antenna are enumerated, respectively. It can be found that the amplitude of ***B*** is larger at a relatively close distance of 50 μm away from the antenna, and the distribution is concentrated directly above the center of antenna. The amplitude of ***B*** decays exponentially with distance, while the distribution of ***B*** diffuses outwards gradually with distance. In order to ensure that the change of magnetic flux density in the air domain can be used to characterize the near-field radiation of the BAW ME antenna, the near-field pattern of an ideal magnetic dipole, as shown in Equation (7), is used for comparison.

(7) $$\left\{ \begin{array}{l} H_{r}=\frac{m_{0}}{2\pi r}j\omega \varepsilon \left[\frac{1}{jkr}+\frac{1}{{\left(jkr\right)}^{2}}\right]e^{-jkr}\cos\theta \\ H_{\theta }=\frac{m_{0}}{4\pi r}j\omega \varepsilon (1+\frac{1}{jkr}+\frac{1}{{\left(jkr\right)}^{2}})e^{-jkr}\sin\theta \\ E_{\varphi }=\frac{m_{0}}{4\pi r}jk(1+\frac{1}{jkr})e^{-jkr}\sin\theta \end{array}\right.,$$

where *k* is the free-wave wavenumber, *H_r_* and *H_θ_* are the magnetic field vectors in the *r* and *θ* directions, respectively, *E_φ_* represents the electric field vectors in the *φ* direction, *ε* is the permittivity of the vacuum, *r* is the propagation distance, and *m*_0_ is the equivalent magnetic dipole moment, which is the volume integral of magnetization in the magnetostrictive layer. It is worth noting that, based on the pointing vector, *H_r_* is a non-radiative standing wave, which indicates the storage and mutual conversion of EM energy. Therefore, the attenuation of *H_r_* with distance and its radiation pattern can be compared with the simulation value to verify the FEA model.

**Figure 7:**
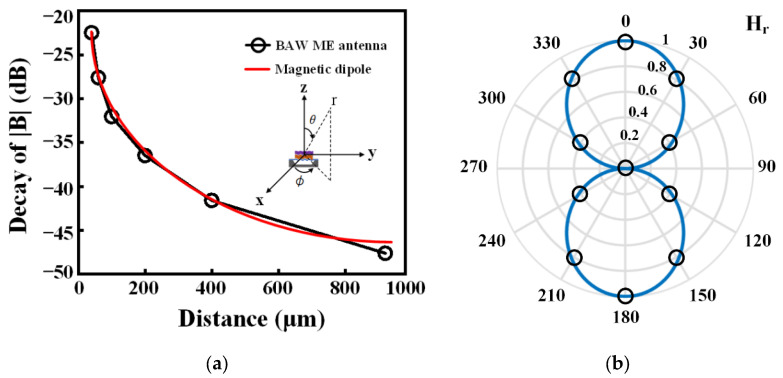
Comparison of near field radiation characteristics between the BAW antenna and the magnetic dipole. (**a**) The magnetic flux density |***B***| decays with distance. The inset shows the BAW ME antenna placed in a spherical coordinate system. (**b**) ***H_r_*** pattern of the magnetic dipole (blue line) and BAW ME antenna (small circle). Each small circle represents the normalized value of simulation |***H_r_***| of different θ at the first-order resonant frequency of 2.6 GHz.

Since the magnetic field data changes dramatically in the near-field region, in order to allow accurate comparison with the simulated data, the decibel (dB) values are used to represent the attenuation of the magnetic flux density. As shown in Figure 7a, the |***B***| decays exponentially with distance and by 45 dB over a distance of 900 μm, which is highly consistent with the calculated results of the ideal dipole. Furthermore, as shown in Figure 7b, by extracting simulation values at different θ and comparing them with the near field of the ideal dipole, it can be found that the magnetic flux density distribution of these discrete positions is equivalent to the radiation pattern of an ideal magnetic dipole. Therefore, this consistency not only proves the feasibility of simulating the near-field radiation of the BAW antenna based on EM perturbations, but also indirectly illustrates that the BAW antenna can be used as a transmitter.

## 4. Conclusions

We demonstrate in principle how to replace the traditional transmitter with one BAW ME antenna to realize the functions of four components. Specifically, a DC magnetic bias and three different modes of a BAW resonator can be excited to use frequency modulation from MF to UHF. Taking the thickness resonant mode as an example, two methods of power amplification are presented, including the increasing of the input voltage and the use of a higher order resonant mode. In particular, a method based on EM perturbation is proposed to evaluate the near-field radiation of the BAW ME antenna, which completely solves the problem that there is no magnetization dynamics model in FEA software. The main characteristic of this simulation is that the variation of magnetic flux density near the antenna is used to replace EM radiation. The simulation results are in good agreement with the radiation theory of magnetic dipole, which not only verifies the model, but also provides a new simulation solution to evaluate the near field distribution of the BAW ME antenna. However, the EM perturbation is different from the EM radiation, so the simulation method in this work cannot be used for modeling the far-field of radiation. The authors are working on the radiation far-field modeling and device preparation of the BAW ME antenna to complete the communication experiment in further work. This work can also provide a simulation idea for the design and analysis of magnetoelectric antennas or transducers.
